# A New Cofilin-Dependent Mechanism for the Regulation of Brain Mitochondria Biogenesis and Degradation

**DOI:** 10.17691/stm2020.12.1.01

**Published:** 2020

**Authors:** T.F. Kovaleva, N.S. Maksimova, P.V. Pchelin, V.I. Pershin, N.M. Tkachenko, M.R. Gainullin, I.V. Mukhina

**Affiliations:** Senior Researcher, Molecular and Cellular Technologies Department, Institute of Fundamental Medicine, Privolzhsky Research Medical University, 10/1 Minin and Pozharsky Square, Nizhny Novgorod, 603005, Russia; PhD Student, Junior Researcher, Molecular and Cellular Technologies Department, Institute of Fundamental Medicine, Privolzhsky Research Medical University, 10/1 Minin and Pozharsky Square, Nizhny Novgorod, 603005, Russia; Laboratory Assistant, Molecular and Cellular Technologies Department, Institute of Fundamental Medicine, Privolzhsky Research Medical University, 10/1 Minin and Pozharsky Square, Nizhny Novgorod, 603005, Russia; Laboratory Assistant, Molecular and Cellular Technologies Department, Institute of Fundamental Medicine, Privolzhsky Research Medical University, 10/1 Minin and Pozharsky Square, Nizhny Novgorod, 603005, Russia; Junior Researcher, Molecular and Cellular Technologies Department, Institute of Fundamental Medicine, Privolzhsky Research Medical University, 10/1 Minin and Pozharsky Square, Nizhny Novgorod, 603005, Russia; Senior Researcher, Molecular and Cellular Technologies Department, Institute of Fundamental Medicine, Privolzhsky Research Medical University, 10/1 Minin and Pozharsky Square, Nizhny Novgorod, 603005, Russia, Researcher, Norwegian PSC Research Center, Department of Transplantation Medicine, Division of Surgery, Inflammatory Diseases and Transplantation, Oslo University Hospital Rikshospitalet, P.O. Box 4950, Nydalen, Oslo, 0424, Norway, Institute of Clinical Medicine, Faculty of Medicine, University of Oslo, P.O. Box 1171, Blindern, Oslo, 0318, Norway; Professor, Director of the Institute of Fundamental Medicine, Privolzhsky Research Medical University, 10/1 Minin and Pozharsky Square, Nizhny Novgorod, 603005, Russia, Head of the Department of Normal Physiology named after N.Y. Belenkov, Privolzhsky Research Medical University, 10/1 Minin and Pozharsky Square, Nizhny Novgorod, 603005, Russia, Professor, Department of Neurotechnologies, Institute of Biology and Biomedicine, National Research Lobachevsky State University of Nizhni Novgorod, 23 Prospekt Gagarina, Nizhny Novgorod, 603950, Russia

**Keywords:** ubiquitin, cofilin, mitochondria, autophagy, PR619, deubiquitinating enzymes

## Abstract

**The aim** Was to study the role of post-translational modifications of cofilin in the regulation of respiration and autophagy in murine brain mitochondria.

**Materials and Methods:**

The experiments were performed with C57BL/6 mice. To obtain cytoplasmic and mitochondrial fractions of the brain tissue, differential centrifugation was used. Expressions of cofilin, phospho-cofilin, K48- and K63-associated chains of ubiquitin, and the autophagy marker LC3B were determined using electrophoresis, immunoprecipitation and Western blot methods. To study the processes of ubiquitination, we used PR619 — the inhibitor of deubiquitinating enzymes. Respiratory activity of brain mitochondria was evaluated using high-resolution fluorespirometry.

**Results:**

Modification of cofilin by non-canonical K63 multiubiquitin chains in the cytoplasm and mitochondria from murine brain was demonstrated. Different levels of phospho-cofilin, cofilin, and its ubiquitinated proteoforms were found. PR619, the inhibitor of deubiquitinating enzymes, affects the expression of phosphorylated and ubiquitinated forms of cofilin in the mitochondria and cytoplasm, at the same time it changes the activity of tissue respiration and mitophagy.

**Conclusion:**

The sensitivity of cofilin to the inhibitor of deubiquitinating enzymes indicates the existence of a new non-catabolic mechanism of cofilin modification, which may be involved in the regulation of mitochondrial functions, specifically, the mitochondrial respiration and autophagy. The data help understand the molecular mechanisms of mitochondrial function in normal and pathological conditions, which may be useful in developing novel methods for the treatment of diseases of the nervous system.

## Introduction

Mitochondria play an important role in many intracellular processes: ATP synthesis, calcium metabolism, apoptosis, nucleotide synthesis, gene expression, and epigenetic changes. The synthesis of ATP occurs with the participation of the electron transport chain coupled with oxidative phosphorylation. The energy transformation in mitochondria is controlled by a number of signaling pathways. Among other factors, the functioning of mitochondria depends on their biogenesis and degradation balance. An important role in this balance is played by mitochondria autophagy (mitophagy), which is able to selectively remove damaged mitochondria and thus maintain normal cellular functions [1]. Cells are able to induce or stop mitophagy in accordance with their energy demands. Accumulation of mitochondria that are not capable of normal functioning may disrupt the respiratory activity and interfere with the expression of genes regulating the mitochondrial network [2]. Moreover, a number of studies showed that autophagy rather than mitophagy could play a crucial role in maintaining the integrity of mitochondrial DNA [3].

Another important pathway of degradation of cellular components is ubiquitination of proteins. Ubiquitination is the covalent attachment of ubiquitin to the accepting lysine exposed on the target protein surface. The ubiquitin-proteasome system is capable of recognizing the proteins that are destined to be destroyed and removed. Resulting from polymerization of several ubiquitin molecules, the so-called multiubiquitin chains are formed. It should be noted that ubiquitination is reversible; a variety of deubiquitinating enzymes are able to detach ubiquitin from its substrates and also cleave the multiubiquitin chains. When studying the process of ubiquitination, it is important to know the sites of the modification, since the binding of ubiquitin to specific lysine residues determines the fate of the target protein and its role in cellular processes. This ubiquitin-associated mechanism regulates most of the cell functions: proliferation and differentiation, DNA repair, intracellular signal transmission, apoptosis, and the immune response [4, 5]. Disruption of the ubiquitin-proteasome system can cause cell damage, and eventually, give rise to malignant neoplasms and neurodegeneration.

It is known that protein degradation driven by the ubiquitin-proteasome system depends on ATP, which is needed for the ubiquitination and for the substrate attachment to the proteasome. In addition, some components of the ubiquitination and deubiquitination systems were identified among the proteins of the outer mitochondrial membrane, e.g., ubiquitin ligase [6]. Notably, a decrease in cellular ATP levels and mild oxidative stress can increase proteasome activity. In pathology, the processes of ubiquitination and subsequent degradation of proteins by the mitochondrial 26S proteasome are impaired.

Mitophagy is also an ubiquitin-associated process. Recent studies have shown that PINK1 kinase (PTEN-induced kinase 1) and E3-ubiquitin ligase (parkin) play an important role in mitophagy [7]. However, the signaling mechanisms of mitophagy are not fully understood. One of the stages of the PINK1–parkin signaling cascade is the remodeling of actin filaments with the participation of LIMK1 kinase and the actin-binding protein cofilin [8]. Parkin can bind to LIMK1 and inactivate it by ubiquitination, which leads to dephosphorylation and activation of cofilin.

Cofilin is known to be involved in various intracellular processes, including the reorganization of the actin cytoskeleton, regulation of gene expression, and triggering programmed cell death [9, 10]. Changes in cofilin structure lead to changes in the respiratory function of cells [11]. At the same time, there is little data on post-translational modifications of cofilin by ubiquitination and its role in the regulation of mitochondrial functions. Nevertheless, the regulatory potential of ubiquitination exceeds all known post-translational modifications and is comparable to the functional role of phosphorylation.

Currently, modification of the ubiquitin-proteasome and ubiquitin-like signaling systems is considered a new promising therapeutic strategy in neurodegenerative diseases.

Studying the role of cofilin and its ubiquitination can contribute in the understanding of the regulation of mitochondrial functions in normal and pathological conditions and the development of new treatments.

**The aim** was to study the role of post-translational modifications of cofilin in the regulation of respiration and autophagy of mouse brain mitochondria.

## Materials and Methods

***Experimental animals*.** The experiments were performed on C57BL/6 mice. The mice were kept in the certified animal facility of the Institute of Fundamental Medicine of the Privolzhsky Research Medical University (Nizhny Novgorod). The environmental conditions and the animal care practice met the requirements of the Guide for the Care and Use of Laboratory Animals (National Research Council, 2011) and the European Convention for the Protection of Vertebrate Animals used for Experimental and Other Scientific Purposes (Strasbourg, 2006); the experimental protocol was approved by the Ethics Committee of the Privolzhsky Research Medical University.

***Preparation of cytoplasmic and mitochondrial fractions of the brain tissue*.** The cytoplasmic and mitochondrial fractions were prepared using differential centrifugation method. After cervical dislocation and decapitation, the brain stem, cerebellum and olfactory components were cut off; the brain hemispheres were washed in an isolation medium (225 mM mannitol; 75 mM sucrose; 5 mM HEPES; 1 mM EGTA; 0.1% BSA; pH 7.4; 4°C) and placed in an incubation medium (110 mM sucrose; 60 mM lactobionic acid; 20 mM taurine; 20 mM HEPES; 10 mM KH_2_PO_4_; 3 mM MgCl_2_; 0.5 mM EGTA; 0.1% BSA; pH 7.2; 4°С). The tissue was ground in a Potter–Elvehjem homogenizer. The resulting homogenate was centrifuged (100 g, 4°C, 10 min). The supernatant was centrifuged again at 9500 g, 4°C for 10 min to obtain the cytoplasmic fraction (supernatant) and mitochondrial fraction (sediment). The pellet was resuspended in the isolation medium without BSA.

The resulting mitochondrial and cytoplasmic fractions were incubated for 2 h with PBS alone in the intact group, 0.25% DMSO (dimethyl sulfoxide) in the control group, and 5 μM PR619 (a deubiquitinating enzyme inhibitor from Abcam, USA) in the experimental group and then used in subsequent experiments.

***Immunoprecipitation, electrophoresis, and immunoblotting*.** After the incubation, the mitochondrial and cytoplasmic fractions of the brain tissue were resuspended in the RIPA lysis buffer at 4°C: 50 mM Tris-HCl; pH 8.0; 500 mM NaCl; 1% NP40; 0.1% SDS; 1 mM NEM; and 0.5 mM of the protease inhibitor cocktail (Sigma, USA) — and were treated with ultrasound. Then the samples were centrifuged at 14,000 g for 15 min at 4°C. Aliquots of each supernatant were used for subsequent experimentation. The protein concentration was made identical in all experimental samples. Immunoprecipitation was performed using anti-cofilin antibodies (Santa Cruz Biotechnology, USA) from the Immunoprecipitation Starter Pack (GE Healthcare, USA) in accordance with the attached instructions.

Protein-containing samples (20 μl/well) and molecular weight markers (ThermoFisher, USA) were loaded on a 12% SDS gel and run for 45 min at 20 mA in a working buffer (0.125 M Tris base; 0.96 M glycine; 0.5% SDS; pH 8.3). The samples were then transferred onto a PVDF membrane in an Immobilon-P system (Merk Millipore, Germany) using a semi-dry tank blot chamber (Bio-Rad Laboratories, USA) in accordance with the manufacturer’s recommendations. Immunoblotting was performed using the SNAP i.d.® 2.0 system (Merk Millipore, USA). All Western blotting procedures were performed at room temperature with the exception of the transfer to PVDF membranes. The membranes were blocked with 1% BSA in PBST (0.02 M PBS containing 0.1% Tween-20; pH 7.4) and then incubated with primary antibodies in PBST with 1% BSA.

The following primary antibodies were used: rabbit polyclonal anti-ubiquitin (Dako, USA); mouse monoclonal anti-ubiquitin (linkage-specific K63) (Abcam, UK); mouse monoclonal anti-cofilin (Santa Cruz Biotechnology); mouse monoclonal anti-β-actin (Santa Cruz Biotechnology); rabbit monoclonal anti-ubiquitin (linkage-specific K48) (Abcam); rabbit monoclonal anti-LC3B (Abcam). Excess primary antibodies were removed from the membranes by washing in PBST. Secondary anti-mouse or anti-rabbit antibodies conjugated to peroxidase (Sigma-Aldrich, Germany) were incubated with the membranes in PBST with 1% BSA for 10 min at room temperature. Excess secondary antibodies were removed by washing the membranes in PBST. Membranes were treated with the ECL reagent (GE Healthcare, USA) and exposed to Amersham Hyperfilm ECL film (Scientific Laboratory Supplies, UK). Detection and quantification of the signal were performed using the Gel Analyzer software.

***Assessment of mitochondrial respiration*.** The rate of oxygen consumption was measured using high-resolution fluorespirometry with the help of an Oxygraph-2k respirometer (Oroboros Instruments, Austria). Incubation medium (2.1 ml) was added to the chambers. The device was calibrated at air saturation and zero oxygen concentration.

At the start of the experiment, mitochondrial respiration was recorded in the absence of exogenous substrates (routine respiration, R). After adding the respiration substrates (5 mM pyruvate, 2 mM malate) but in the absence of ADP (adenosine diphosphate), the respiration rate reflected the non-phosphorylating state of the respiratory chain associated with mitochondrial complex I. The rate of respiration during oxidative phosphorylation associated with complex I was measured after adding 1.25 mM ADP, and that associated with complexes I and II — after adding 10 mM succinate (P). Then, the ATP synthase inhibitor oligomycin (2 ng/ml) was added to identify the respiration in the non-phosphorylating state with the participation of complexes I and II (L). The maximum respiration rate associated with complexes I and II was estimated after the sequential addition of the CCCP — the carbonyl cyanide m-chlorophenylhydrazone protonophore (E). The maximum activity of the respiratory chain associated with complex II was measured after the addition of 0.5 μM rotenone, an inhibitor of complex I (Rot). After adding 2.5 μM antimycin A (an inhibitor of complex III), the oxygen consumption rate was no more associated with the respiratory chain of mitochondria, but was due to other oxidative reactions. To assess the function and regulation of the respiratory chain of mitochondria, we calculated the following ratios: R/E, L/E, P/E, Rot/E, P/L (respiratory control). The primary data were processed using the DatLab 5.0 software and normalized per 1 mg of mitochondrial protein. The protein content was determined by the Bradford method. The rate of oxygen consumption (O_2_) was expressed in picomoles per second/1 mg of mitochondrial protein.

***Statistical analysis*.** Data are presented as mean ± standard error of the mean (M±SEM). The significance of differences between the experimental groups was determined using Student’s t-test. Differences were considered statistically significant at p≤0.05.

## Results

***Post-translational modifications of cofilin in the cytoplasm and mitochondria of the murine brain tissue***

**Figure 1:**
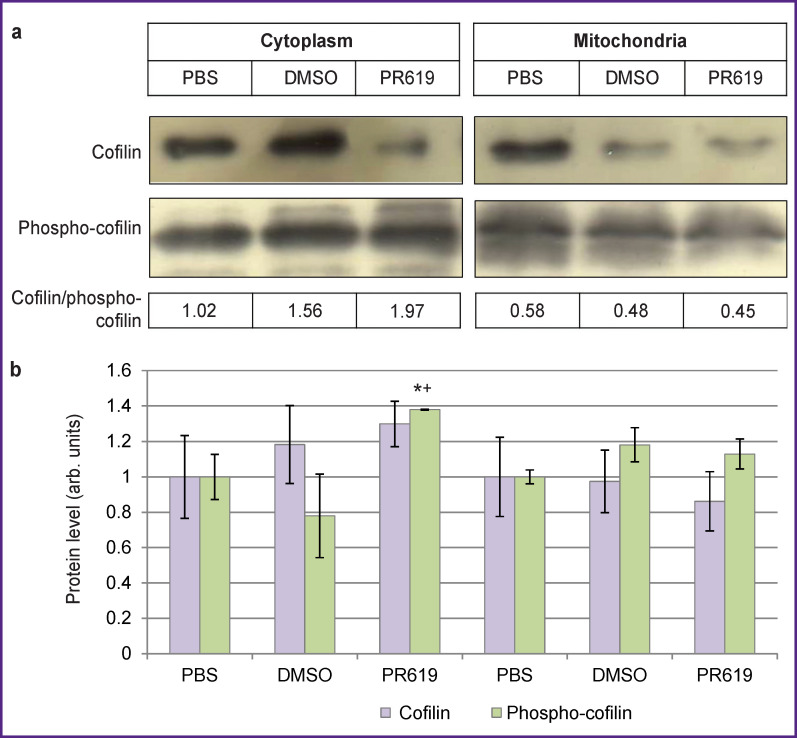
Expression of cofilin and phospho-cofilin in the cytoplasm and mitochondria from murine brain tissue in the presence of the deubiquitinating enzyme inhibitor PR619: (a) detection of monomeric cofilin and phospho-cofilin by Western blotting; (b) change in the level of monomeric cofilin and phospho-cofilin under PR619; the values are normalized per levels of these proteins in the intact group (PBS). * Statistically significant differences from the intact group values, p≤0.05; **^+^** from the control, p≤0.05

*Expression of phospho-cofilin and cofilin.* Western blotting was used to study the intracellular level of phospho-cofilin and cofilin. Figure 1 shows expression levels of phospho-cofilin and monomeric cofilin (17 kDa) in the mitochondrial and cytoplasmic fractions of mouse brain tissue. We found that the PR619, inhibitor of deubiquitinating enzymes, caused a decrease in the level of monomeric cofilin in the mitochondria (as compared to the intact and control groups), as well as a 1.3-fold increase in the level of cofilin in the cytoplasm (compared with the intact group). In the presence of PR619, expression of phospho-cofilin in the cytoplasm was significantly higher than the respective values in the intact and control groups; some increase was also found in the mitochondria. Incubation of mitochondria with PR619 led to a decrease in the cofilin/phospho-cofilin ratio in the experimental group, which was indicative of cofilin inactivation.

*Cofilin ubiquitination in the cytoplasm and mitochondria*. These processes were studied using immunoprecipitation and Western blot analysis. The Western blotting with anti-cofilin antibodies revealed the presence of monomeric (17 kDa) and medium-molecular weight forms (proteoforms) of cofilin in lysates of the cytoplasm and mitochondria, as well as in the immunoprecipitates.

**Figure 2:**
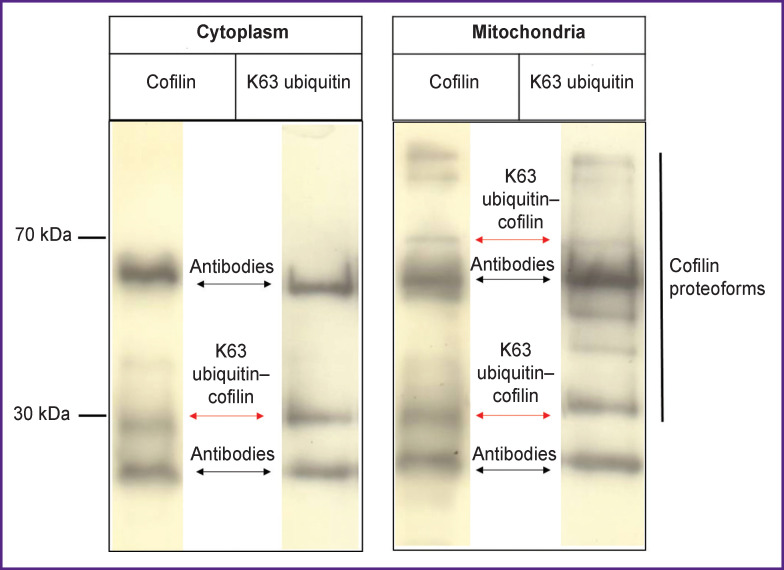
Western blot analysis of cofilin ubiquitination in the cytoplasm and mitochondria from murine brain tissue Ubiquitination in the cofilin immunoprecipitates was identified with antibodies against K63-associated ubiquitin chains

At the next step, we studied the type of ubiquitin chains associated with cofilin in the cytoplasmic and mitochondrial fractions of murine brain tissue. To this end, we analyzed cofilin immunoprecipitates with antibodies against Lys48- and Lys63-associated ubiquitin chains (K63 ubiquitin). Lys48-modified cofilin proteoforms were detected in both the mitochondrial and cytoplasmic fractions. In addition, the medium-molecular weight forms of cofilin (30 and 70 kDa) were found to have specific cross-immunoreactivity with K63 chains of ubiquitin, thus indicating a modification of cofilin by K63 multiubiquitin chains (Figure 2).

**Figure 3:**
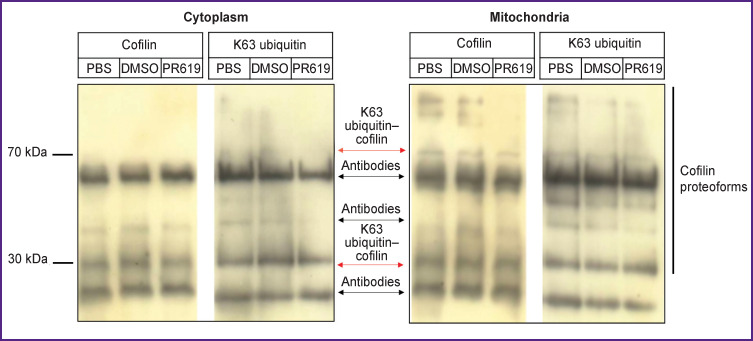
Western blot analysis of cofilin levels in the cytoplasm and mitochondria from murine brain tissue in the presence of the deubiquitinating enzyme inhibitor PR619

Further quantification of the PR619 effect showed a 1.5-fold increase in the level of cofilin modified by K63 ubiquitin multichains (30 kDa) in the cytoplasm as compared to the intact group with no difference vs. the control group. At the same time, PR619 did not change the level of medium molecular weight proteoforms of cofilin in the mitochondria (Figure 3).

**Figure 4:**
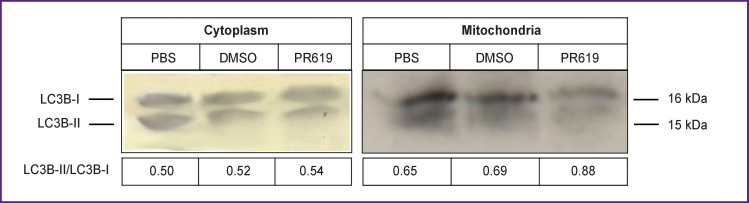
Western blot analysis of LC3B levels in the cytoplasm and mitochondria from murine brain tissue in the presence of the deubiquitinating enzyme inhibitor PR619

***The study of autophagy in the cytoplasmic and mitochondrial fractions of brain tissue.*** We found that in the presence of PR619, the level of LC3B-I decreased and the level of LC3B-II increased in the mitochondrial fraction; as a result, the LC3B-II/LC3B-I ratio increased in the mitochondria (Figure 4). In the cytoplasm, the ratio of LC3B-II/LC3B-I did not change.

**Figure 5:**
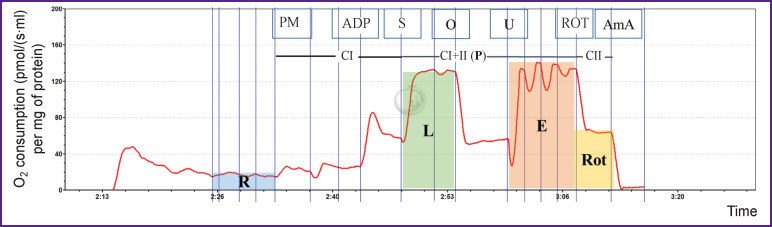
Respirometry record of mitochondria from murine brain tissue in the presence of the deubiquitinating enzymes inhibitor PR619: *PM* — pyruvate + malate; *ADP* — adenosine diphosphate; *S* — succinate; *O* — oligomycin; *U* — uncoupling in the presence of CCCP (carbonyl cyanide m-chlorophenylhydrazone); *ROT* — rotenone; *AmA* — antimycin A. *CI* — oxygen consumption rate after addition of pyruvate, malate, and ADP; *CI+II* (*P*) — oxygen consumption rate after succinate is added; *CII* — oxygen consumption rate with the participation of complex II of the respiratory chain. *R* — routine respiration on endogenous substrates; *L* — respiration in the non-phosphorylating state with the participation of complexes I and II; *E* — the maximum respiration rate after titrating with CCCP; *Rot* — oxygen consumption rate after the addition of rotenone — the inhibitor of the respiratory complex I of mitochondria

***Analysis of mitochondrial respiration*.** To study the mitochondrial respiration, high-resolution fluorespirometry was used (Figure 5).

The following estimated ratios were used to evaluate the function of the respiratory chain: R/E, L/E, P/E, R/P, P/L (respiratory control), and Rot/E (see the Table). In the presence of PR619, the R/E ratio increased about 1.4-fold as compared with the intact and control groups. A 2-fold increase in the ratio of the complexes I and II-associated respiration (with the mixture of substrates) to the maximum respiration (P/E) was detected in the presence of PR619; no changes were found in the intact or control groups.

**Table:** Functional states of the respiratory chain in mitochondria from murine brain tissue (M±SEM)

Group	R/E	L/E	P/E	R/P	P/L	Rot/E
PBS (intact)	0.10±0.03	0.19±0.04	0.92±0.01	0.11±0.04	4.69±0.02	0.29±0.07
0.25% DMSO (control)	0.10±0.01	0.17±0.02	0.92±0.05	0.11±0.05	5.26±0.21*	0.28±0.06
5 μM PR619 (experimental)	0.14±0.02*^+^	0.39±0.05*^+^	0.10±0.01*^+^	0.08±0.02	4.65±0.16^+^	0.60±0.08*^+^

* Statistically significant differences as compared with the intact group, p≤0.05; ^+^ with control, р≤0.05.

Significantly lower ratios of the respiration rate with endogenous substrates to respiration with the substrate mixture (R/P) and a significant increase in the Rot/E ratio in mitochondria treated with PR619 were found.

## Discussion

Changes in energy metabolism (the transition from oxidative phosphorylation to glycolysis and *vice versa*) depend on remodeling of the mitochondrial network, including the removal of damaged mitochondria and the biogenesis of new ones [12]. Moreover, disruption of the mitochondrial electron transport chain and ATP synthesis have an impact on autophagy and mitophagy.

In the present work, post-translational modifications of cofilin and its role in the mitochondrial functions were studied using murine brain mitochondria. We established the presence of both phosphorylated and dephosphorylated forms of cofilin in the cytoplasmic and mitochondrial fractions of brain cells. According to the results, the inactive phosphorylated form of cofilin predominates in the mitochondria of the intact group. Recent studies have shown that mitochondrial division and mitophagy depend on the assembly/disassembly of actin filaments, regulated by the actin-depolymerization activity of cofilin [13].

Phosphorylation — dephosphorylation is considered the main mechanism regulating the cofilin activity. Dephosphorylated cofilin can bind to G-actin and then translocate to mitochondria, which changes the actin cytoskeleton, causes mitochondrial dysfunction, provokes a release of cytochrome C, and apoptosis [14]. Cofilin-mediated Bax translocation is thought to be a key event in the excitotoxic death of neurons [15].

In the next step, we studied the process of cofilin ubiquitination. Ubiquitination is one of the most important mechanisms of post-translational modification of proteins. The regulatory potential of ubiquitination exceeds all known post-translational modifications and is comparable to phosphorylation as far as the control over cell function is concerned. Specifically, the ubiquitin system is involved in regulating the cell cycle, intracellular signal transmission, apoptosis, and DNA repair, whereas ubiquitination plays a crucial role in the pathogenesis of diseases [4, 5].

The analysis of cofilin immunoprecipitates with antibodies against Lys48- and Lys63-associated ubiquitin chains indicated the presence of medium molecular weight cofilin proteoforms modified in the lysine 48 and 63 positions both in the mitochondria and cytoplasm of brain cells. In the present study, non-canonical modification of cofilin by K63 multiubiquitin chains has been demonstrated for the first time in the mitochondria of nerve cells. These results are consistent with data on cofilin modified by ubiquitin chains in epithelial cells [16].

The decrease in the cofilin/phospho-cofilin ratio in the murine brain mitochondria treated with PR619, the inhibitor of deubiquitinating enzymes, shows cofilin inactivation. Under the same PR619 treatment though, the level of cofilin modified by K63 multiubiquitin chains (30 kDa) increased in the cytoplasm (with some decrease in the mitochondria), which indicated a redistribution of the medium-molecular weight proteoforms of cofilin between the cytoplasm and mitochondria. The sensitivity of cofilin to PR619 may reflect a new non-catabolic mechanism of cofilin regulation.

The obtained data suggest that the intracellular deubiquitinating enzymes closely are related to the mechanism of control over cofilin proteoforms. It is possible that the modification of cofilin by K63 multiubiquitin chains can trigger additional intracellular processes. According to the literature, K63-associated ubiquitin chains can be a signal of degradation of ubiquitinated proteins by autophagy [17]. Specifically, the C-terminal ubiquitin-binding domain of the p62 protein can bind both Lys48- and Lys63-associated ubiquitin chains with a higher affinity for Lys63 chains. Both autophagy and the ubiquitin-proteosome system are the two main mechanisms for the degradation of intracellular components. In addition, it has been shown that Lys63-associated ubiquitin chains activate the NF-κB transcription factor, DNA repair, immune response, removal of damaged proteins and mitochondria, and splicing/translation of mRNA.

It is known that K63 ubiquitination plays a key role in the proteosome-associated protein degradation upon the formation of branched K48/K63 chains of ubiquitin. Notably, the deubiquitinating enzymes specific for the K63-associated chains of ubiquitin block this pathway. Our results showed an increase in the LC3B-II/LC3B-I ratio in the mitochondria, which might indicate an activation of the mitophagy process. The physiological role of deubiquitinating enzymes in the cell is to control the ubiquitination processes and maintain stability of proteins. Hyper- or hypofunction of these enzymes can lead to cell pathology [18]. For example, USP30 deubiquitinase is able to inhibit cell apoptosis and stop mitophagy mediated by the parkin protein (E3 ligase) [19].

According to Seiberlich et al. [20], inhibition of deubiquitinating enzymes leads to the accumulation of ubiquitinated proteins, the formation of protein aggregates and the subsequent inhibition of the ubiquitin-proteasome system. Using an oligodendroglial cell line (OLN-t40), the authors showed that inhibition of deubiquitinating enzymes led to activation of autophagy. Thus, the activation of mitophagy by PR619 may partially compensate for the malfunction of the ubiquitin-proteasome system and indicate coordination between these degradation systems.

Based on the obtained data, it can be assumed that the protein ubiquitination correlates with mitochondrial dysfunction. Functional and structural damage to mitochondria in various diseases leads to impaired energy metabolism, decreased ATP levels, an increased production of reactive oxygen species and premature death of nerve cells. In this work, using the method of high-resolution respirometry, a change in mitochondrial respiration was detected in the presence of PR619 — the broadly specific inhibitor of deubiquitinating enzymes. The increase in the R/E ratio in the presence of PR619 indicated that the respiratory chain of mitochondria was functioning at a close to maximum velocity.

Previously, using confocal fluorescence microscopy and fluorescence lifetime imaging microscopy [21], we showed an increase in mitochondrial respiration (compared to the rate of glycolysis) in a murine brain hippocampal cell culture treated with PR619; the present results are consistent with the previous ones. The increase in the P/E ratio indicates the inability of mitochondria to use the substrates of complexes I and II for oxidative phosphorylation. Likewise, the low R/P ratio in the presence of PR619 also indicates an insufficient ATP synthesis activity. The increase in the Rot/E ratio indicates a dysfunction of the mitochondrial complex I in the presence of PR619. At the same time, the increased R/E ratio may reflect a decrease in the membrane potential of the mitochondria. It is known that a disturbance of the membrane potential can trigger the processes of mitophagy and autophagy.

Thus, the effect of deubiquitinating enzyme inhibitors on mitochondrial respiration and mitophagy suggests the involvement of the ubiquitin-proteasome system in the regulation of these two processes. Redistribution of cofilin proteoforms between the cytoplasm and mitochondria with a change in the mitochondrial functions indicates the involvement of cofilin and its post-translational forms in the regulation of mitochondrial biogenesis and degradation.

## Conclusion

The biogenesis and degradation of mitochondria are significant factors of cell physiology and pathology. The sensitivity of cofilin to the deubiquitinating enzyme inhibitor suggests the existence of a new, non-catabolic mechanism that controls the structure and function of cofilin. Such a mechanism may be involved in the regulation of mitochondrial functions, respiration, and autophagy. By targeting the ubiquitin-proteasome system and ubiquitin-like signaling systems it is possible to find new therapeutic solutions. Understanding the mechanisms controlling the mitochondrial functions is important for developing novel strategies for the prevention and treatment of diseases of the nervous system.
